# Interactive E-learning module in pharmacology: a pilot project at a rural medical college in India

**DOI:** 10.1007/s40037-013-0081-0

**Published:** 2013-09-27

**Authors:** Nitin Gaikwad, Suresh Tankhiwale

**Affiliations:** 1 Department of Pharmacology, All India Institute of Medical Sciences, Tatibandh, G. E. Road, Raipur, 492099 Chhattisgarh India; 2 Jawaharlal Nehru Medical College, Datta Meghe Institute of Medical Sciences (Deemed University), Sawangi (Meghe), Wardha, 442004 Maharashtra India; 3 Center for Health Professional Education and Research, Jawaharlal Nehru Medical College, Datta Meghe Institute of Medical Sciences (Deemed University), Sawangi (Meghe), Wardha, 442004 Maharashtra India; 4 Flat No. 303, 2nd Floor, Type 5A, AIIMS Residential Complex, Kabir Nagar, Raipur, 492099 Chhattisgarh India

**Keywords:** E-learning, Pharmacology, Active learning, Self-learning, PowerPoint

## Abstract

Many medical educators are experimenting with innovative ways of E-learning. E-learning provides opportunities to students for self-directed learning in addition to other advantages. In this study, we designed and evaluated an interactive E-learning module in pharmacology for effectiveness, acceptability and feasibility, with the aim of promoting active learning in this fact-filled subject. A quasi-experimental single-group pre-test/post-test study was conducted with fourth-semester students of the second professionals course (II MBBS), selected using non-probability convenience sampling method. An E-learning module in endocrine pharmacology was designed to comprise three units of interactive PowerPoint presentations. The pre-validated presentations were uploaded on the website according to a predefined schedule and the 42 registered students were encouraged to self-learning using these interactive presentations. Cognitive gain was assessed using an online pre- and post-test for each unit. Students’ perceptions were recorded using an online feedback questionnaire on a 5-point Likert scale. Finally, focused group discussion was conducted to further explore students’ views on E-learning activity. Significant attrition was observed during the E-learning activity. Of the 42 registered students, only 16 students completed the entire E-learning module. The summed average score of all three units (entire module) was increased significantly from 38.42 % (summed average pre-test score: 11.56/30 ± 2.90) to 66.46 % (summed average post-test score: 19.94/30 ± 6.13). The class-average normalized gain for the entire module was 0.4542 (45.42). The students accepted this E-learning activity well as they perceived it to be innovative, convenient, flexible and useful. The average rating was between 4 (agree) and 5 (strongly agree). The interactive E-learning module in pharmacology was moderately effective and well perceived by the students. The simple, cost-effective and readily available Microsoft PowerPoint tool appealed to medical educators to use this kind of simple E-learning technology blended with traditional teaching to encourage active learning among students especially in a rural setup is attractive.

## Introduction

Today is the era of information technology. E-learning, which is defined as the use of internet technologies to deliver a wide array of solutions that enhance knowledge and performance, has become an important component of today’s teaching–learning process in higher education institutes [1, 2].

Indian Universities are taking on this newer methodology, mostly in a blended format. UNESCO defines knowledge society as a society which values creation and sharing of new knowledge, so that the new knowledge can be applied for the well-being of its people [3]. Educators are of the opinion that wider participation of students in this knowledge society can be facilitated by enhancing their competencies in information and communication technologies [4]. Previous studies have also reported that E-learning can be used as an active learning strategy which promotes self-directed learning. This innovative learning can have a big impact on the arena of self-directed learning [5].

The Medical Council of India’s (MCI) Vision 2015 Document has put the emphasis more on non-didactic teaching–learning methodology. In addition, this document proposes newer teaching methodology in the form of E-learning. The Regulation on Graduate Medical Education, 1997 Document of the MCI, New Delhi, states that the broad goal of the teaching of undergraduate students in pharmacology is to inculcate a rational and scientific basis of therapeutics [6]. Students must develop an understanding of concepts in pharmacology, and acquire knowledge about drugs and therapeutics, as well as the skills to select and prescribe medicines based on clinical conditions and pharmacological properties, efficacy, safety, suitability and cost of medicines for common clinical conditions of national importance. E-learning provides an opportunity for students to improve the learning experience by ease of access, greater interactivity, and individual choice concerning the pace and mix of learning. At the same time, it also offers advantages for teachers such as improved distribution of learning content, ease of update, standardization, and tracking of learner activities [7].

With the aim of encouraging active learning among pharmacology undergraduate medical students of the second professional level at the rural medical college of central India, we designed interactive E-learning modules. The objectives of the present study were:

- To measure the effectiveness of the interactive E-learning module in pharmacology
- To assess the perceptions of students about the interactive E-learning module in pharmacology
- To assess the acceptability and feasibility of E-learning in undergraduate medical students at rural set-up

## Materials and methods

### Ethics committee approval

The study was conducted after obtaining approval from the institutional ethics committee.

### Study design and sample

This was a quasi-experimental single-group pre-test/post-test study conducted at the rural teaching Medical College of Central India, with fourth-semester students in the second professional MBBS course (II MBBS students). Students were selected by the non-probability convenient sampling method. Students were encouraged to register voluntarily for this E-learning project by sending their details (name, roll number and mobile number) to the principal investigator’s email address. Students’ email ID and mobile number were used to communicate the teaching–learning and assessment programme during the project. A total of 42 students registered at the beginning of the project.

### Study material

#### E-learning module

An informal discussion was conducted with the students, pharmacology faculties, and faculties of the Centre for Health Professions Education and Research (CHPER) to decide the topic for the E-learning activity. Faculties and students suggested that the topic should be from the ‘must know’ portion of the pharmacology syllabus and should have already been taught by traditional didactic instruction. Based on these suggestions, it was decided to prepare an E-learning module on endocrine pharmacology.

Three themes from endocrine pharmacology were identified and an E-learning module in endocrine pharmacology was prepared comprising three units: Unit 1—corticosteroids, Unit 2—thyroid and anti-thyroid drugs, and Unit 3—oestrogens, progestin and hormonal contraceptives.

Each unit comprised pre-test, learning resource material in the form of interactive PowerPoint presentations, post-test and feedback questionnaire. The total duration for each unit was 7 days, which includes 5 days for the E-learning session. Thus, the total duration for entire E-learning module (all three units) was 21 days.

#### Interactive PowerPoint presentations

Interactive PowerPoint presentations were developed in Microsoft office PowerPoint 2007, using action buttons and hyperlinks according to the support guidelines available on the Microsoft office website. The following measures were taken to make PowerPoint presentations interactive:

- Each presentation was divided into several sections.
- The presentation was set up to run at a kiosk. This mode disables mouse click and arrow keys on the keyboard to advance to the next slide.
- Action buttons and hyperlink were added to move to the next slide, previous slide, home and various sections.
- After each section, a test question (multiple-choice question) slide was inserted. The test question slide was designed in such way that the only correct answer to the question will take the student to the next section. Running in a kiosk feature, thus, prevents students from viewing the correct answer and hence, prevents direct access to the next section.
- The presentation was saved in the PPSX file format. The advantage of this file format is that on double-click the presentation will not open in an editable format. A PPSX file automatically opens in a slide show mode rather than a normal view.
- Learning points were added to emphasize the important points on a particular topic.

The standard textbooks on pharmacology recommended in the medical undergraduate pharmacology curriculum were used as standard reference for preparing presentations. Learning objectives for each presentation were defined and were included at the beginning of each presentation. Learning objectives and the text content of the PowerPoint presentation were validated from the subject expert in pharmacology and faculty from CHPER. A validated interactive PowerPoint presentation of each unit was uploaded on the website according to a predefined schedule of E-learning sessions.

#### Website designing

A website was designed on Google Sites with the objective of uploading learning resource material (Interactive PowerPoint Presentations) [8]. The complete web address of the website is https://sites.google.com/site/pharmacelearning/. Appropriate navigation keys were given on the home page of the website for easy access to the students.

### Pre-test, post-test and students’ feedback questionnaire

The pre-test and post-test prepared for each unit comprised ten multiple-choice questions. Questions in the pre- and post-test were similar. Multiple-choice questions were linked to the learning objectives of each unit to ensure content validity. The maximum possible score in each test was 10 and thus, for the entire module it was 30. Tests were pre-validated.

A feedback questionnaire comprising six items, five open-ended and one close-ended, was prepared for each unit to record students’ perceptions on the utilization of learning resource material. In addition, the overall feedback questionnaire comprising ten items, eight close-ended and two open-ended, was prepared for the entire E-learning module to record students’ perceptions on E-learning activity in pharmacology. A 5-point Likert scale (1 = strongly disagree to 5 = strongly agree) was used to record responses of close-ended items. For open-ended items, students were asked to contribute their free comments. All feedback questionnaires were pre-validated.

After validation, tests and feedback questionnaires were completed online using the SurveyMonkey tool available on the net for creating online surveys (SurveyMonkey, 2012). The basic (free) version of SurveyMonkey was used to create online tests and the feedback questionnaire. A personalized web link for each test and feedback was sent to each registered student at their email IDs according to a predefined schedule of E-learning sessions.

### Methodology (E-learning sessions)

Informed consent was taken from the registered students before their participation in the study. Two contact sessions (face-to-face) were conducted, one at the beginning and the second at the end of E-learning sessions. In the first contact session, the students were briefed about the study and the study objectives were explained.

**Fig. 1:**
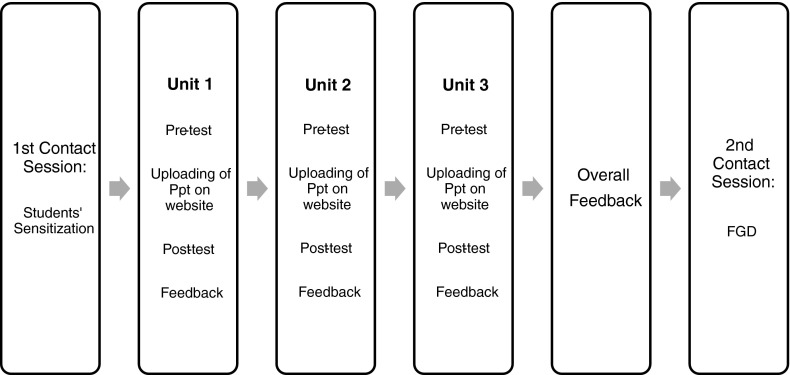
Schematic presentation of e-learning activity

After the first contact session, three E-learning sessions were taken sequentially as outlined in Fig. 1. Each session was of 7-day duration and the entire module was completed in 21 days.

At the end of the E-learning sessions, a second contact session in the form of a focused group discussion (FGD) was conducted. For FGD, ten students were selected based on their responses to the feedback questionnaire, which included dropout students as well. FGD script was prepared and the entire discussion was video-recorded with the prior permission of participating students.

### Data analysis and statistics

The E-learning project was evaluated using Kirkpatrick’s Evaluation Model [9]. In the Level 1 evaluation, students’ perceptions were evaluated using the feedback questionnaire and FGD. Responses to close-ended items recorded on a 5-point Likert scale were expressed as percentages and the average rating score was calculated. Responses to open-ended items were analyzed qualitatively. Responses were classified into various themes and categories.

In the Level 2 evaluation, the pre-test and post-test score were compared for cognitive learning gain of each unit separately as well as cumulatively for those participants who completed the entire E-learning module (all three units). Missing data were not included in the analysis. The analysis was restricted to only those students who had completed both the pre- and post-test. The pre- and post-test score was added for all three units and the average was calculated to determine the cumulative score. The cumulative score was only determined for those students who had completed both the pre- and post-test of all the three units (*N* = 16). Student’s paired *t* test was used to compare pre- and post-test scores. A *p* value <0.05 was considered significant. Absolute learning gain (%post-test score − %pre-test score) and relative learning gain (%post-test score − %pre-test/%pre-test score) were calculated. The effectiveness of the intervention was evaluated using class-average normalized gain [g = (%post-test score − %pre-test score)/(100 − %pre-test score)] [10]. A class-average normalized gain (g) of 0.3, i.e. 30 %, was considered significant according to Hake’s criteria for the effectiveness of an educational intervention [10, 11].

## Results

### Demographic details and Internet use pattern

Of the registered students, 69.05 % were female and 30.95 % were male. Of the students, 42.86 % participants were dependent on the digital library facility on-campus or internet café, 33.33 % used their personal internet connection and the rest used either mobile internet or a friend’s connection to access E-learning modules and online assessment.

### Students’ response rate

**Fig. 2:**
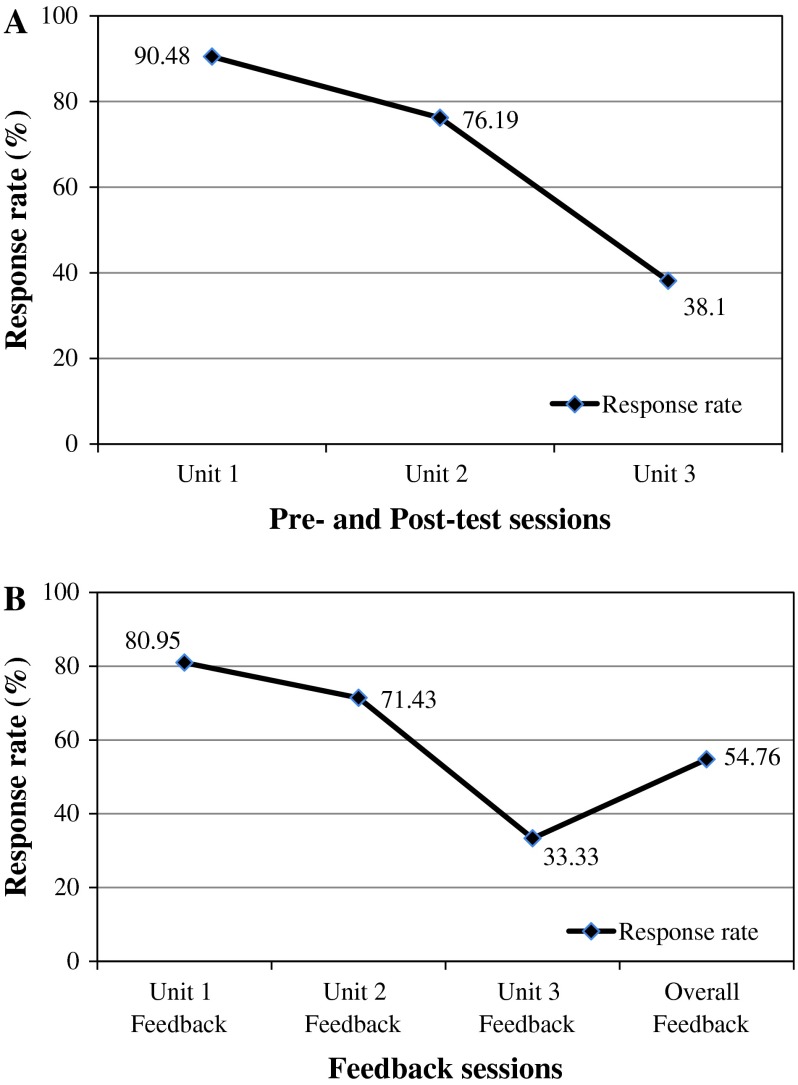
**A** Response rate: pre-test and post-test. **B** Response rate: feedback

The response rate for online pre-test/post-test and feedback dropped from Unit 1 to Unit 3. The response rate for online pre- and post-test decreased from 90.48 for Unit 1 (*N* = 38) to 76.19 for Unit 2 (*N* = 32) and 38.10 for Unit 3 (*N* = 16). Similarly, the response rate for online feedback was decreased from 80.95 for Unit 1 (*N* = 34) to 71.43 for Unit 2 (*N* = 30) and 33.33 for Unit 3 (*N* = 14). The response rate for overall feedback was 54.76 (*N* = 23) Fig. 2a, b.

### Cognitive learning gain

The average post-test scores improved significantly in all three units after educational intervention in the form of interactive PowerPoint presentations shared via the website. The absolute learning gain was 29.47, 40.31 and 22.50 % in Unit 1, 2 and 3, respectively. The relative gain was 60.22, 127.72 and 64.29 % in Unit 1, 2 and 3, respectively. Class-average normalized gain for Unit 1, 2 and 3 was 0.5773 (57.73 %), 0.5890 (58.90 %) and 0.3462 (34.62 %), respectively.

Only 16 students undertook the pre- and post-tests of all three units. For these students, the average test score increased significantly from 38.42 % (summed pre-test score of three units: 11.56/30 ± 2.90) to 66.46 % (summed post-test score of 3 units: 19.94/30 ± 6.13). The absolute learning gain was 27.92 % whereas relative learning gain was 72.43 %. The class-average normalized gain was 0.4542 (45.42 %) (Table 1).

### Students’ feedback

#### Students’ perception on utilization of learning resource material

The Cronbach’s alpha, a measure of internal consistency of questionnaire items, for Unit 1, 2 and 3 was 0.812, 0.784 and 0.718, respectively. The students perceived that the learning resource material was informative, useful and innovative. The rating score ranged between 4 and 5, i.e. agree and strongly agree (Table 2).

**Table 1:** Pre-test, post-test scores and cognitive learning gain

Score/gain	Unit 1 (*N* = 38)	Unit 2 (*N* = 32)	Unit 3 (*N* = 16)	Cumulative (*N* = 16)
Pre-test score (%pre-test)	4.89 ± 2.22 (48.95)	3.16 ± 1.72 (31.56)	3.50 ± 1.63 (35)	11.56 ± 2.90 (38.54)
Post-test score (%post-test)	7.84 ± 2.20* (78.42)	7.19 ± 2.33* (71.88)	5.75 ± 3.55† (57.50)	19.94 ± 6.13* (66.46)
Absolute learning gain	29.47	40.31	22.50	27.92
Relative learning gain	60.22	127.72	64.29	72.43
Class-average normalized gain	57.73	58.90	34.62	45.42

Values of pre-test and post-test score are represented as mean ± SD (%)

Maximum score for each unit: 10, maximum score for e-learning module (cumulative): 30

Absolute gain, relative gain and class-average normalized gain expressed as percentage

* *p* < 0.001 using Student’s paired *t* test (pre-test score vs post-test score)

† *p* = 0.019 using Student’s paired t-test (pre-test score vs post-test score)

**Table 2:** Students’ perceptions on utilization of learning resource material

Sr. no.	Statements	Students’ rating		
		Unit 1 (*n* = 34)	Unit 2 (*n* = 30)	Unit 3 (*n* = 14)
1	Improved understanding of topic	4.50 ± 0.56	4.57 ± 0.57	4.43 ± 0.85
2	Informative and useful	4.59 ± 0.50	4.40 ± 0.56	4.36 ± 0.63
3	User friendly	4.41 ± 0.66	4.23 ± 0.68	4.21 ± 0.70
4	Good learning experience	4.47 ± 0.61	4.37 ± 0.67	4.43 ± 0.65
5	Will enhance performance in examination	4.44 ± 0.61	4.40 ± 0.62	4.43 ± 0.65

Values of students’ rating are represented as mean ± SD

#### Students’ perception on E-learning activity

The Cronbach’s alpha for overall feedback questionnaire on E-learning activity was 0.736. The students’ opinion ranged between score 4 to score 5, i.e. between agree to strongly agree. Average rating score ranged from 4.09 to 4.61 whereas the mode was observed to be 4 for initial four items and 5 for next four items (Table 3).

**Table 3:** Students’ perception on E-learning activity

Sr. No.	Statements	SD	D	N	A	SA	Students’ rating	Mode
1	Learning atmosphere (non-classroom teaching) during E-learning sessions motivated me as a learner	0 (0.00)	0 (0.00)	1 (4.35)	11 (47.83)	11 (47.83)	4.43 ± 0.59	4
2	E-learning sessions enhanced my self-learning ability	0 (0.00)	0 (0.00)	1 (4.35)	13 (56.52)	9 (39.13)	4.35 ± 0.57	4
3	E-learning suits my learning style	0 (0.00)	1 (4.35)	5 (21.74)	11 (47.83)	7 (30.43)	4.09 ± 0.73	4
4	E-learning sessions complemented traditional learning through didactic lectures	0 (0.00)	0 (0.00)	3 (13.04)	13 (56.52)	7 (30.43)	4.17 ± 0.65	4
5	E-learning could replace few didactic lectures in pharmacology	0 (0.00)	0 (0.00)	3 (13.04)	7 (30.43)	12 (52.17)	4.30 ± 0.88	5
6	E-learning could be incorporated in pharmacology curriculum	0 (0.00)	0 (0.00)	1 (4.35)	10 (43.48)	12 (52.17)	4.48 ± 0.59	5
7	E-learning could be recommended for other subjects as well	0 (0.00)	0 (0.00)	1 (4.35)	7 (30.43)	15 (65.22)	4.61 ± 0.58	5
8	E-learning in pharmacology was innovative and useful	0 (0.00)	0 (0.00)	0 (0.00)	9 (39.13)	14 (60.87)	4.61 ± 0.50	5

*N* = 23

Values in parentheses indicate percentage

Likert scale: *SD* strongly disagree, *D* disagree, *N* neutral, *A* agree, *SA* strongly agree

#### Analysis of free comments

The free comments in response to open-ended questions were analyzed qualitatively and categorized as barriers, facilitating factors, and suggestions (Table 4).

**Table 4:** Barriers, facilitating factors and suggestions for E-learning session

Barriers	Facilitating factors	Suggestions/specific comments
Slow internet connection	Simple, compact and easy to understand presentations	Digital library facilities at hostels
Non-compatible presentations with mobile phones	No binding of place, pace and time for learning	Frequently asked questions should be mentioned at the end of the module
	Pre-test and post-test	Cover entire pharmacology syllabus under this E-learning program
	Test questions in presentations	Test questions should be provided for download for future references.
	Important points highlighted	Number of questions should be increased for tests.

#### Analysis of FGD

Students’ responses recorded in FGD were categorized into five main themes, viz learning resource material (Interactive PowerPoint), E-learning activity, interactivity, online assessment and IT facilities on-campus. These themes were again classified into various categories and students’ responses were summarized under these categories (Table 5).

**Table 5:** Summary of FGD

Sr. no.	Themes of FGD	Subcategories and comments
1	Learning resource material (interactive Ppt)	Positive comments
		Ease of access, test after each section, important learning points, easy language
		Negative comment
		No animation or video
		Suggestions
		Use of animation and videos, clinical cases, ADR photographs
		Pedagogic value of E-learning module
		Flexibility of time and place, self-assessment component, learner’s autonomy
2	E-learning activity	Motivating factors
		Modern way of learning, innovative, exam-oriented topic, readymade notes
		Best part of E-learning activity
		No binding of place, pace and time, non-threatening learning environment, self-assessment
		E-learning vs classroom teaching
		Non-threatening learning environment, active learning, time conserving
		Suggestions for implementation
		Complimentary rather than replacement of traditional teaching, faster internet with Wi-Fi
3	Interactivity	Suggested modalities for interaction
		Face-to-face interaction in addition to online interaction through email and GoogleGroups
4	Online assessment	Positive comment
		Easy to mark and submit, compatible with mobile phones, tests are like quiz and motivated for learning
		Negative comment
		Scores not displayed after submission of test
		Suggestions
		Questions in pre- and post-test should be different with same difficulty level, progressive disclosure of questions
5	IT facilities on-campus	Problems and suggested solutions
		Slow/suspended internet connection—faster internet connection with Wi-Fi
		Digital library timing not suitable—extend digital library timing beyond daily teaching schedule
		No computer terminals and internet at hostel—two to three computer terminals at hostel with internet connection

## Discussion

Commonly used instructional strategies to teach pharmacology are didactic lectures, tutorials, practical sessions, seminars and symposiums. Although medical teachers are inculcating a culture of self-directed learning among medical students to facilitate active learning, we cannot overlook the role of technology for this cause. A literature search showed that researchers used various forms of E-learning tools such as streaming video, multimedia, web-based interactive module, and Moodle [12–15]. In pharmacology, researchers also used various tools such as E-learning courses to improve prescribing skills, delivering concepts in clinical pharmacology and therapeutics through E-learning, integration of pathophysiology into pharmacology through a web-based E-learning course [7, 16–18].

In this E-learning project, we sought to employ technology in the form of interactive PowerPoint presentations shared via the website during E-learning sessions and assessed through online tests, to promote self-directed learning among medical undergraduates.

Behaviouristic theory states that learning content needs to be split into several chunks or learning units. In addition, learning units need to be represented in a specific order which defines the learning path. We divided the contents of each presentation into several sections. The learning path was defined through navigation keys such as action buttons and hyperlinks to several sections. To enhance the interactivity of presentation, we added learning points and test questions after each section. Knowles [19] believed adults did not normally pursue learning simply for the sake of learning, but because they need to immediately apply what they were learning to life situations. Knowles proposed that immediate application of knowledge enhances learning. Bargellini mentioned that self-assessment helps learners to steer themselves during the learning path and measure their knowledge of a topic [20]. This was determined using test questions. We argue that while using PowerPoint as an E-learning tool, medical educators should use test questions linking two sections. Learning points after every few slides acted as checkpoints for interactions. These checkpoint interactions in the form of learning points helped in managing the learner’s cognitive load as well as in engaging the learner in problem-solving activities, rather than passively digesting the course content [21].

The pre-/post-test was used to determine cognitive gain during E-learning sessions. The threats to internal validity of pre-test/post-test results such as history, testing, Hawthorne effect and halo effect, do not affect two groups or multigroup design, but these extraneous variables may affect results of educational research involving evaluation of knowledge gain out of educational intervention in the single group study design [10, 22]. As the pre-/post-test was administered on different occasions with a gap of 4–5 days, the possibility of history, maturation and testing effect cannot be ruled out. Many studies in computer-aided learning have used a single session as the unit of analysis [23]. However, in this E-learning module, we conducted three E-learning sessions and we used all these three sessions as the unit of analysis. This longitudinal approach can mitigate a potential problem of the first approach, where the limited duration of the experiments may be partly responsible for the lack of convergent findings [24].

The class-average normalized gain (g) has been used as a measure of effectiveness of an educational intervention in previous studies [25]. The class-average normalized gain is independent of the study group’s pre-test level of knowledge. Previous research in physics has shown that average pre-test scores (%pre) are nearly independent of the pre-test score, being dependent primarily on the effectiveness of the instruction [25]. The class-average normalized gain is categorized as follows: 0.1–0.29 low gain, 0.3–0.69 medium gain and 0.7–1.0 high gain [11, 25]. In our study, we observed a class-average normalized gain of 0.5773 (57.73 %), 0.5890 (58.90 %), 0.3462 (34.62) for Unit 1, 2 and 3, respectively. In addition, when we calculated cumulative class-average normalized gain for the entire module, it was 0.4542 (45.42 %). A class average normalized gain of more than 0.3 but <0.69, as defined criteria by Hake, indicates that educational intervention in the form of E-learning module in pharmacology was moderately effective.

A significant dropout in response rate for the online pre-/post-test and feedback was observed. However, differential attrition, which is considered a threat to internal validity of results, is a problem for two- or multi-group designs but not for the single-group design [22]. Hence, attrition in this study will not affect the results of this study. Attrition in E-learning courses is a global problem. Previous studies have also suggested that dropout rates are higher in online courses as compared with traditional classroom courses [26, 27]. Hart [28] discussed that various factors are associated with the persistence of students with online courses such as satisfaction with online learning, a sense of belonging to learning community, motivation, peer and family support, time management skills and increased communication with the instructor. However, in FGD, we tried to find the reasons behind this significant decrease in response rate in our study. The important reasons for higher dropouts were poor internet connectivity, less interaction via emails, students’ engagement in exam preparation, different learning style, computer illiteracy and hindrance in accessing learning resource material.

The E-learning concept in pharmacology was well perceived by the students as evident from the pre-/post-test analysis, feedback and FGD. For the first time, we introduced the concept of E-learning in a medical college situated in a rural part of central India. In this study, we explored PowerPoint, a readily available tool of Microsoft Corporation, as a learning resource material. The downloadable format of these presentations helped students to put presentations on CDs and Pendrive and to take them anywhere they wanted. This disappearance of boundaries of learning further boosted their self-learning ability and enhanced their efficacy and self-confidence. In addition, we used the free website from Google Sites and the SurveyMonkey tool. All these tools are free and cost-effective. They do not need any special software. A basic knowledge of the computer, PowerPoint presentations and internet operations would be good enough for the educators to develop E-learning modules. Thus, such types of E-learning modules are easy to prepare and access. It is a myth among teachers that E-learning will require costly electronic equipment, software and a server. With available resources, we can prepare simple E-learning modules and can share them via free internet sites.

Nevertheless, we also faced a few challenges, the most important being internet connectivity. The internet is a lynchpin of any E-learning project. Most of the participants did not have a personalized internet connection and this poses a big challenge. For successful implementation of E-learning programmes, computer and internet access to students is essential. A few students faced difficulty in downloading PowerPoint presentations on their mobile due to an incompatible format. Hence, while preparing any E-learning module, it is essential for moderators to determine its compatibility on different devices for better access for the end-users. Other limitations of this study were the single-group study design, and use of the simple convenience sampling method. It will be imperative to compare E-learning with traditional teaching and blended teaching methods in a randomized controlled study in the future. It will also be worthwhile to explore the role of newer electronic gadgets such as Smartphones, iPads, and Tablets in E-learning activity.

We did this pilot project with the objective of exploring the feasibility of implementing E-learning. Digital library facility at campus favours the implementation of E-learning. In his study at the National Open University of Nigeria, Otubelu (2011) [29] also suggested E-learning through digital libraries. However, for the implementation phase, we will need to consider various factors. First, we need to consider the computer literacy among medical students as well as faculties, as we have to implement this on a large-scale basis. Feeling the need for computer literacy among medical undergraduates, the MCI has already announced its strategy to enhance this through induction of a Foundation Course approach in the undergraduate curriculum. Second, we require better IT facilities and students and faculty sensitization for E-learning. Third, from an administrative standpoint, we will have to look at the costs involved and electronic equipment such as the computer, modem and internet connection required for a smooth implementation. In order to enhance the efficacy of E-learning environment, teachers and students must be given ample support in terms of training, equipment and time resources.

If E-learning is to be implemented successfully, we need to consider three dimensions of self-directed learning: personal attributes which include intrinsic motivation, learning style, resourcefulness and robust cognitive strategy; the learning process which incorporates learner autonomy that works as a continuum from didactic lectures where the lecturer has 100 % control to the learner taking control of his learning in E-learning; and learning context which includes learning environment, the ‘anywhere anytime’ characteristics of E-learning. Considering these dimensions, a blended learning approach with traditional teaching can be implemented which provides deeper learning.

## Conclusion

Curriculum innovation in the form of an interactive E-learning module in pharmacology was moderately effective and well perceived by the students. Students’ acceptability for this innovation was further strengthened by their suggestion for inclusion of E-learning sessions in pharmacology and other subjects as well. The use of readily available computer tools such as Microsoft PowerPoint and Internet, rather than specialized software, and the cost-effectiveness strongly appeal to medical educators; the use of this kind of simple E-learning technology blended with traditional teaching to encourage active learning among student is attractive. Nevertheless, it will be worthwhile to further explore the scope of E-learning, especially in medical colleges in rural areas.

## Essentials

- Pre-test-post-test analysis showed significant improvement in the post-test score in all units of E-learning module.
- Cumulative class-average normalized gain for the entire E-learning module was 0.4542 (45.42 %), which is more than 30 % indicating moderate effectiveness of E-learning module.
- Students perceived the E-learning module in pharmacology to be very useful, informative and user friendly.
- Simple computer tools such as PowerPoint can be used as E-learning tools to encourage self-directed learning.

## References

[1] Rosenberg MJ (2001). E-learning: strategies for delivering knowledge in the digital age.

[2] Seluakumaran K, Jusof FF, Ismail R, Husain R (2011). Integrating an open-source course management system (Moodle) into the teaching of a first-year medical physiology course: a case study. Adv Physiol Educ.

[3] Bindé J. Towards knowledge societies: UNESCO world report. UNESCO reference works series. 2005. Paris: UNESCO. p. 220. http://unesdoc.unesco.org/images/0014/001418/141843e.pdf. Accessed 9 May 2012.

[4] Tan SC, Divaharan S, Tan L, Cheah HM. Self-directed learning with ICT: Theory Practice and Assessment [monograph online], 2011. Singapore: Ministry of Education. http://ictconnection.edumall.sg. Accessed 9 May 2012.

[5] Ozuah PO (2002). Undergraduate medical education: thoughts on future challenges. BMC Med Educ.

[6] Salient features of Regulations on Graduate Medical Education, 1997 (Internet). 1997. Published in part iii, section 4 of the Gazette of India dated 17th May 1997. http://www.mciindia.org/RulesandRegulations/GraduateMedicalEducationRegulations1997.aspx. Accessed 22 January 2012.

[7] Maxwell S, Mucklow J (2012). E-learning initiatives to support prescribing. Br J Clin Pharmacol.

[8] Google Sites makes creating and sharing a group website easy [internet]. Google Inc., California, United States. http://www.google.com/sites/help/intl/en/overview.html. Accessed 30 Dec 2011 .

[9] The Official Site of the Kirkpatrick Model [internet]. Kirkpatrick’s Partner, Atlanta, GA. http://www.kirkpatrickpartners.com/Home/tabid/38/Default.aspx. Accessed 3 Jan 2012.

[10] Colt HG, Davoudi M, Murgu S, Zamanian Rohani N (2011). Measuring learning gain during a one-day introductory bronchoscopy course. Surg Endosc.

[11] Prather EE, Rudolph AL, Brissenden G (2009). Teaching and learning astronomy in the 21st century. Phys Today.

[12] Bridge PD, Jackson M, Robinson L. The effectiveness of streaming video on medical student learning: a case study [internet]. Med Educ Online 2009;14:11. http://med-ed-online.net/index.php/meo/article/view/4506/4686. Accessed 2 April 2012. 10.3885/meo.2009.Res00311 PMC2779626 20165525

[13] Davids MR, Chikte U, Halperin ML (2011). Development and evaluation of a multimedia E-learning resource for electrolyte and acid-base disorders. Adv Physiol Educ.

[14] Oeffner F, Schäfer C, Fritz B, et al. Interactive E-learning courses in human genetics: Usage and evaluation by science and medical students at the faculty of medicine [internet]. GMS Zeitschrift für Medizinische Ausbildung 2011;28(3):Doc38. http://www.egms.de/static/pdf/journals/zma/2011-28/zma000750.pdf. Accessed 23 June 2012. 10.3205/zma000750 PMC3159195 21866240

[15] Schilling K, Wiecha J, Polineni D, Khalil S (2006). An interactive web-based curriculum on evidence based medicine: design and effectiveness. Fam Med.

[16] Maxwell SR (2012). How should teaching of undergraduates in clinical pharmacology and therapeutics be delivered and assessed?. Br J Clin Pharmacol.

[17] Gordon M, Chandratilake M, Baker P (2011). Improved junior paediatric prescribing skills after a short E-learning intervention: a randomized controlled trial. Arch Dis Child.

[18] Tse MM, Lo LW (2008). A web-based E-learning course: integration of pathophysiology into pharmacology. Telemed J E Health.

[19] Knowles MS (1984). Androgogy in action.

[20] Bargellini ML, Caiaffa E, Casadei G, Coletti S, Puccia L. e-Dissemination of Geographic Information Science: *SIGEO* course [internet]. http://itcnt05.itc.nl/agile_old/Conference/estoril/papers/105_M.L.%20Bargellini.pdf. Accessed 29 Aug 2012.

[21] Greitzer FL. A cognitive approach to student-centered E-learning [internet]. A research paper presented at Human Factors and Ergonomics Society 46th Annual Meeting, Sept 30–Oct 4, 2002. http://pro.sagepub.com/content/46/25/2064.abstract. Accessed 31 July 2012.

[22] Johnson B, Christensen L, editors. Chapter 8 Validity of research results. In: Educational Research: Quantitative, Qualitative and Mixed Approaches [internet]. 2003. http://www.southalabama.edu/coe/bset/johnson/lectures/lec8.htm. Accessed 27 July 2012.

[23] Reeves TC (1993). Pseudoscience in computer based instruction: the case of learner control research. J Comput Based Instr.

[24] Zhang D, Zhou L, Briggs RO, Nunamaker JF. Instructional video in E-learning: Assessing the impact of interactive video on learning effectiveness [internet]. Information & Management 2006; 46:15–27. Available from: http://www.qou.edu/arabic/researchProgram/eLearningResearchs/instructional.pdf. Accessed 12 Aug 2012.

[25] Hake RR (1998). Interactive-engagement vs traditional methods: a six-thousand-student survey of mechanics test data for introductory physics courses. Am J Phys.

[26] Diaz DP. Online drop rates revisited. The technology source archives. May/June 2002. http://www.technologysource.org/article/online_drop_rates_revisited. Accessed 22 Aug 2012.

[27] Kreideweis J (2005). Indicators of success in distance education. Comput Inform Nurs.

[28] Hart C. Factors associated with student persistence in an online program of study: a review of the literature [internet]. J Interac Online Learn 2012;11(1):19–42. http://www.ncolr.org/jiol/issues/pdf/11.1.2.pdf. Accessed 26 Aug 2012.

[29] E-learning through digital libraries: the case of National Open University of Nigeria [internet]. Library Philosophy and Practice 2011. http://digitalcommons.unl.edu/cgi/viewcontent.cgi?article=1647&context=libphilprac. Accessed 25 Aug 2012.

